# Decellularization of human dermis using non-denaturing anionic detergent and endonuclease: a review

**DOI:** 10.1007/s10561-014-9467-4

**Published:** 2014-08-28

**Authors:** Mark A. Moore, Brian Samsell, Glenna Wallis, Sherry Triplett, Silvia Chen, Alyce Linthurst Jones, Xiaofei Qin

**Affiliations:** Institute of Regenerative Medicine, LifeNet Health, 1864 Concert Drive, Virginia Beach, VA 23453 USA

**Keywords:** Allograft, Homograft, Dermis, Decellularized, Acellular dermal matrix, MatrACELL

## Abstract

Decellularized human dermis has been used for a number of clinical applications including wound healing, soft tissue reconstruction, and sports medicine procedures. A variety of methods exist to prepare this useful class of biomaterial. Here, we describe a decellularization technology (MatrACELL^®^) utilizing a non-denaturing anionic detergent, N-Lauroyl sarcosinate, and endonuclease, which was developed to remove potentially immunogenic material while retaining biomechanical properties. Effective decellularization was demonstrated by a residual DNA content of ≤4 ng/mg of wet weight which represented >97 % DNA removal compared to unprocessed dermis. Two millimeter thick MatrACELL processed human acellular dermal matrix (MH-ADM) exhibited average ultimate tensile load to failure of 635.4 ± 199.9 N and average suture retention strength of 134.9 ± 55.1 N. Using an in vivo mouse skin excisional model, MH-ADM was shown to be biocompatible and capable of supporting cellular and vascular in-growth. Finally, clinical studies of MH-ADM in variety of applications suggest it can be an appropriate scaffold for wound healing, soft tissue reconstruction, and soft tissue augmentation.

## Introduction

Decellularization technology has been utilized to remove cellular components in a variety of soft tissues including cardiovascular allograft and human dermal matrix to produce bio-implants for clinical application. The objectives of the decellularization process are to remove potentially immunogenic material and provide a biocompatible scaffold for host cellular and vascular in-growth (Norton and Babensee 2009). Following decellularization, the remaining extracellular matrix can also be used as a scaffold for tissue engineering (Pellegata et al. 2013). Decellularized cardiovascular tissue has been applied in a variety of in vivo applications (Ketchedjian et al. 2005a, b; Hopkins et al. 2009; Elkins et al. 2001a, b; Sievers et al. 2003; Simon et al. 2003; Hawkins et al. 2003; Bechtel et al. 2003, 2005; Kasimir et al. 2006; Steinhoff et al. 2000; Cebotari et al. 2002). Similarly, human acellular dermal matrix (ADM) has been used for wound healing, soft tissue reconstruction, and sports medicine applications. Specifically, human ADM has been reported to be used clinically for repair of rotator cuff tears (Wong et al. 2010; Snyder and Bone 2007; Barber et al. 2008; Burkhead et al. 2007; Bond et al. 2008; Dopirak et al. 2007), during which the dermal matrix is typically used to augment a repair procedure in order to provide biomechanical strength as well as support directed healing. Also, Achilles and quadriceps tendon augmentation procedures using human ADM are reported with satisfying clinical outcomes (Wilkins 2010; Lee 2007, 2008; Barber et al. 2006). In addition, human ADM is commonly used for soft tissue reconstruction procedures including primary, staged, and revision breast reconstruction (Sbitany et al. 2009; Nahabedian 2009; Salzberg 2006) and hernia repair (Kapfer and Keshen 2006; Albo et al. 2006; Candage et al. 2008; Mitchell and Cima 2011). Moreover, human ADM is widely used in the treatment of chronic wounds such as diabetic foot ulcers (Winters et al. 2008; Randall et al. 2008; Brigido et al. 2004).

In particular, one decellularization technology, MatrACELL^®^ (US Patent 6,743,574 (2004)) (LifeNet Health, Virginia Beach, VA), has been applied to human pulmonary patches, which received 510(k) clearance from the FDA and has been in clinical use since 2009 (Lofland et al. 2012). The same MatrACELL technology is also applied to human dermis and the resultant ADM is referred to here as MatrACELL processed human acellular dermal matrix (MH-ADM). The processing, properties, and potential applications of MH-ADM are reviewed herein.

## The MatrACELL decellularization and sterilization process

The MatrACELL decellularization process was developed to minimize the impact of processing reagents on biomechanical and biochemical properties of the tissue while still removing cellular components (US Patents 6,734,018 (2004); 7,338,757 (2008)). MatrACELL- processed tissue is rendered acellular in a solution of non-denaturing anionic detergent (N-Lauroyl sarcosinate, NLS), recombinant endonuclease, and antibiotics (including Polymixin B, Vancomycin and Lincomycin). Following decellularization, the tissue is thoroughly rinsed to remove the decellularization reagents. Next, the bio-implant is treated with a water replacing agent, such as glycerol (US Patents 6,293,970 (2001); 6,544,289 (2003); 6,569,200 (2003); 7,063,726 (2006)), prior to final packaging of the tissue. This allows room temperature storage and rapid preparation by the end user. Finally, the bio-implant is terminally sterilized with low temperature, low dose gamma irradiation (Moore et al. 2004). This final step results in a Sterility Assurance Level (SAL) of 1 × 10^−6^ as anticipated for a medical device, while also inactivating viruses (Moore 2012). The entire process retains biomechanical and biocompatible (Qin et al. 2008) properties of the MH-ADM.

## Preclinical evaluation of MH-ADM

MH-ADM was assessed via analytical methods, biomechanical testing, and in vivo analysis. Representative study results are presented in this review. Results from original data sets are not intended to be generalizable, but add novel information to be considered in totality with the other studies presented here.

### Histological analysis overview

**Fig. 1:**
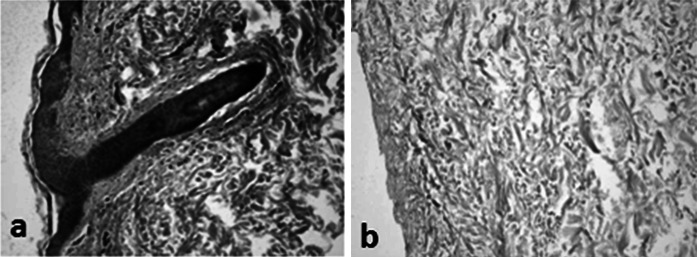
Histological analysis of human skin tissue. **a** prior to and **b** after MatrACELL processing. Hemotoxylin and eosin (H&E) staining shows nuclear material (blue/purple staining). Note the presence of stained nuclear material prior to decellularization (**a**) in contrast to the lack of nuclear material in the tissue after the MatrACELL process in MH-ADM (**b**). (Data on file at LifeNet Health). (Color figure online)

**Fig. 2:**
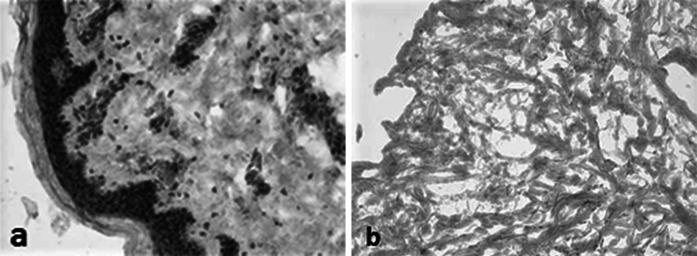
Major Histocompatibility Complex I (MHC-I) staining of human skin tissue **a** prior to and **b** after the MatrACELL process. MHC-I staining was used to detect cells and cellular remnants. Note the presence of brick-red stained MHC-I positive cells prior to decellularization (**a**) in contrast to the absence of MHC-I staining in the tissue after the MatrACELL process (**b**). (Data on file at LifeNet Health). (Color figure online)

The MH-ADM process is designed to remove cellular remnants from tissue. Evidence of decellularization by histological analysis is demonstrated by the absence of cell nuclei (Fig. 1) and cell membrane components such as major histocompatibility complex I (MHC-I) (Fig. 2). MHC-I plays a vital role in cell immunity, but is considered an undesirable cellular remnant in tissue implantation (Steinmetz 1986). This is due to MHC-I’s capability of activating recipient’s immune response, specifically CD8+ T cells, against the newly implanted tissue, and, thus, its removal is desirable for clinically implantable materials.

### Analysis of DNA residuals

The DNA content in the MH-ADM was quantified using Quant-iT™ PicoGreen^®^ dsDNA assay kits (Life Technologies, Inc., Carlsbad, CA) and calculated as ng/mg tissue weight (Table 1) using one tissue sample each from three donors (n = 3). Compared to unprocessed dermis, at least 97 % of DNA content was removed through processing.

**Table 1:** DNA content for dermis before and after the MatrACELL decellularization process quantified at minimal, nominal, and maximal processing parameters

Condition	Average DNA in dermis (ng/mg wet weight)	Avg standard deviation	Average DNA in MH-ADM Dermis (ng/mg wet weight)	Avg standard deviation	Reduction (%)
Minimum	118.18	19.23	1.67	0.16	98.58
Nominal	107.36	13.89	2.78	0.14	97.41
Maximum	132.98	12.24	1.76	0.12	98.68

**Fig. 3:**
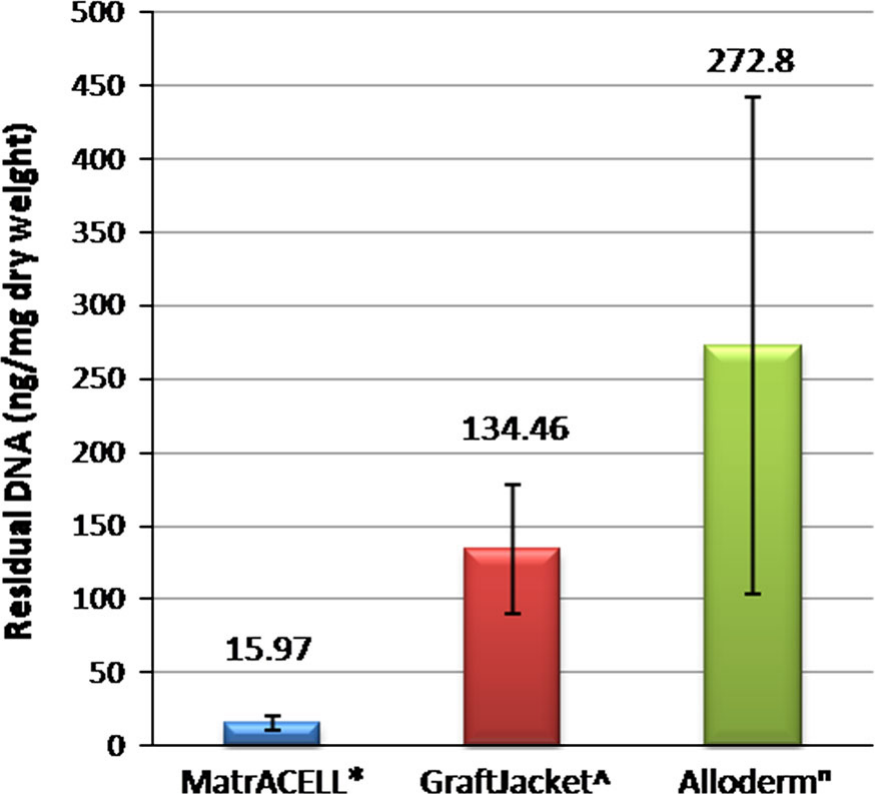
Comparison of residual DNA content in three ADM. All DNA content results are presented as ng/mg of dry weight of material. Please note this is not a side-by-side experiment, rather a comparison to the literature. However, all values are represented as ng/mg dry weight of tissue, and the method of detection was identical. *Asterisk* MH-ADM (Data on file at LifeNet Health, n = 3 as 1 sample each from 3 donors) and values reported in the literature for: ^ GraftJacket (Derwin et al. 2010), ^n^ AlloDerm (Choe and Bell 2001)

All DNA content results are presented as ng/mg of wet weight of material (Data on File, n = 3 as 1 sample each from 3 donors, LifeNet Health). Note the substantial DNA reduction at all parameters as compared to the non-decellularized dermis control. Also note, in contrast to Fig. 3, these values are presented per wet weight of dermis

In contrast, the post-processing DNA content of two other commercially available decellularized human tissues, AlloDerm^®^ (LifeCell Corporation, Branchburg, NJ) and GraftJacket™ (Wright Medical Technology, Inc., Arlington, TN), as reported in the literature (Derwin et al. 2006; Choe and Bell 2001) were much greater than that found in MH-ADM (Fig. 3). The content of DNA in MH-ADM was measured to be 15.97 ± 4.8 ng/mg dry weight. Using a similar PicoGreen dsDNA Assay (Molecular Probes), DNA content in GraftJacket was reported to be an average 134.6 ± 44.0 ng/mg dry weight (Derwin et al. 2006) and 272.8 ± 168.8 ng/mg tissue for AlloDerm (Choe and Bell 2001).

### Biomechanical testing overview

**Fig. 4:**
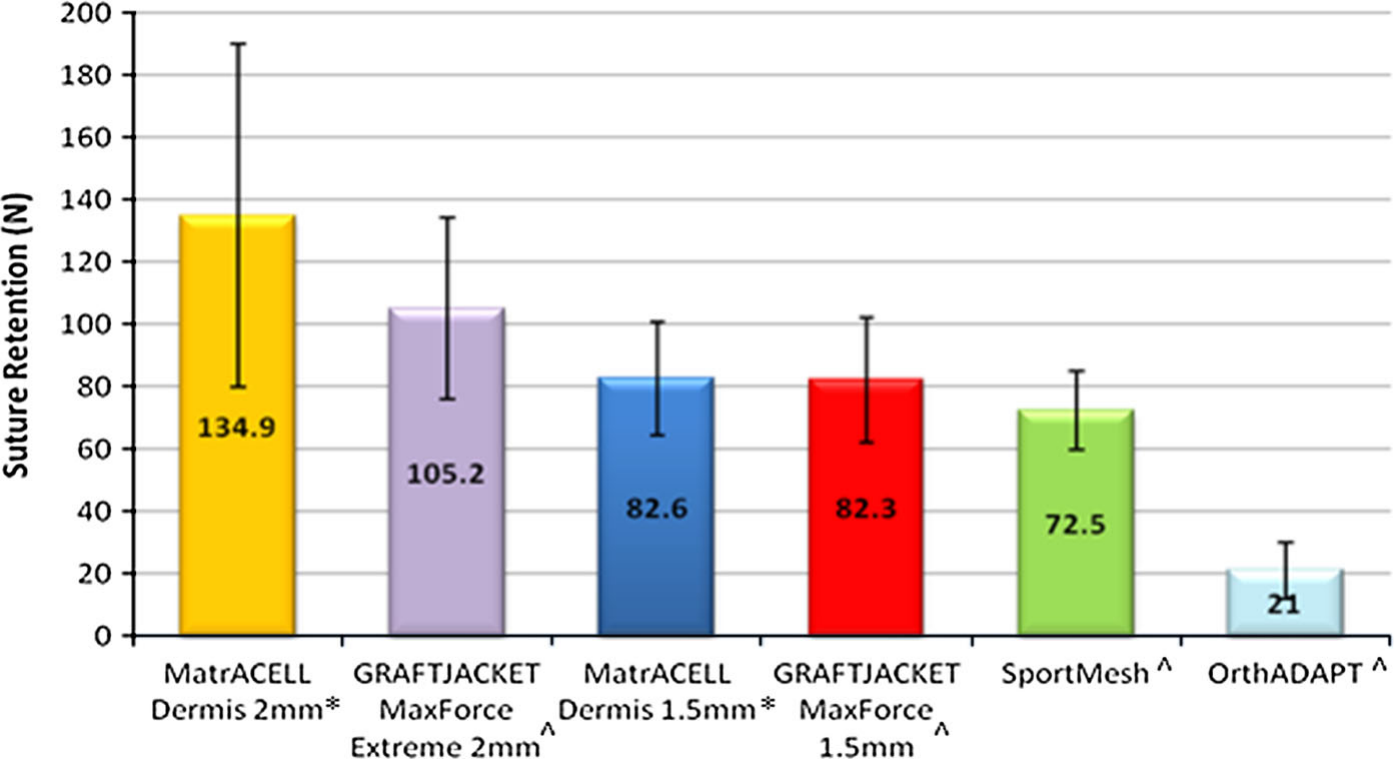
Suture pullout strength comparison of two thickness MH-ADM (2 mm* and 1.5 mm*) compared with two thickness GRAFTJACKET and other surgical mesh products (^). Data generated for MatrACELL Dermis and all other materials, respectively was generated at different points in time; however, the exact same methods, fixtures, material testing machine, and facility was used for both studies. *Asterisk* Data on File at Arthrex (n = 5), ^ Barber and Aziz-Jacobo (2009)

**Fig. 5:**
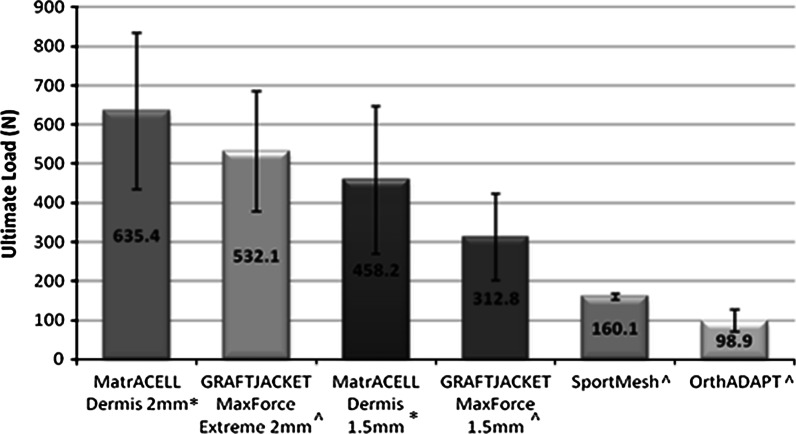
Ultimate load to failure comparison of two thickness MH-ADM (2 and 1.5 mm*) is compared with two thickness GRAFTJACKET and other surgical mesh products (^). Data generated for MatrACELL Dermis and all other materials, respectively was generated at different points in time; however, the exact same methods, fixtures, material testing machine, and facility was used for both studies. *Asterisk* Data on File at Arthrex, ^ Barber and Aziz-Jacobo (2009)

Dependent on the intended use, the biomechanical properties of MH-ADM may be of clinical significance, especially in potentially load bearing applications such as tendon or rotator cuff augmentation. To assess, suture retention strength was measured as the force needed to pull out a ‘simple vertical stitch’ of Arthrex No.2 FiberWire^®^ (Arthrex, Inc., Naples, FL) passed through the tissue 5 mm from the edge (Barber and Aziz-Jacob 2009). In addition, ultimate load was determined by pressure clamping two ends of a single layer of material and elongating to failure. The suture retention and ultimate load tests were conducted on two different thicknesses of MH-ADM (2 and 1.5 mm) and GraftJacket as well as SportsMesh™ (Biomet Sports Medicine, LLC, Warsaw, IN) and OrthoDAPT™ (Pegasus Biologics, Inc., Irvine, CA) (Figs. 4, 5). The mechanical testing focused on different types of soft-tissue augmentation materials and no control was included. Typically, higher suture retention strength and ultimate tensile strength were found in the thicker tissue. While the types of failure for suture pullout were not noted, the ultimate tensile tests primarily failed at the midsection. As demonstrated, the biomechanical integrity of MH-ADM compared favorably with other materials used in soft-tissue augmentation procedures.

Moreover, the biomechanical properties of MH-ADM was investigated (Beitzel et al. 2012) in rotator cuff augmentation procedures performed on randomly assigned cadaveric fresh frozen shoulders. Note that MH-ADM is branded as ArthroFLEX^®^ (Arthrex, Inc., Naples, FL) in this publication. The study compared MH-ADM interposed between the bone and tendon as well as placed on top of the repair. Double-row repairs without augmentation served as the control. No significant difference was found in ultimate load to failure between the control group (348.9 ± 98.8 N) and the group with interposed MH-ADM (469.9 ± 148.6 N). However, the group with MH-ADM placed on top had significantly higher load to failure (575.8 ± 22.6 N; *P* = 0.025) than the non-augmented control (438.9 ± 98.8 N).

Additionally, the biomechanical strength of intact scapholunate ligaments and the ligaments reconstructed with 1.5 and 1.0 mm thick MH-ADM (also described as ArthroFLEX^®^ in this published study by Eshan et al. 2012) was measured in cadaveric tests. While the intact ligament serving as the control failed mid-substance during tensile testing, the 1.0 mm MH-ADM reconstructed ligament failed at the suture-dermal matrix interface and the 1.5 mm MH-ADM reconstructed ligament failed at the suture-bone anchor interface. The authors concluded that the positive results warrant further clinical investigation for using MH-ADM as a potential treatment for chronic scapholunate instability.

### Small animal study: in vivo results

**Fig. 6:**
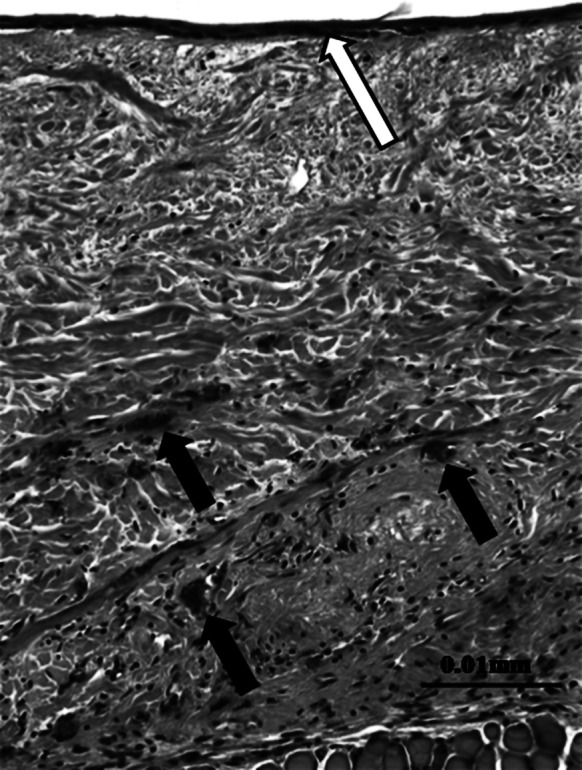
Hematoxylin and eosin staining of MH-ADM explants using nude mouse skin excisional model. The implant was in place for 16 days prior to excision and analysis. Note the presence of new blood vessels (*black arrows*) and epithelial layer (*open arrow*) (data on file at LifeNet Health). (Color figure online)

MH-ADM was tested using a nude mouse skin excisional wound model (n = 3). No control of unprocessed dermis was included as this was intended only as an assessment of processed dermis. In this study, a portion of full thickness skin was excised from the back of a nude mouse and replaced with MH-ADM and covered with a dressing. After 16 days, the implanted dermis was removed and examined histologically for cellularity and inflammation. Normal fibroblast infiltration was observed through the entire thickness of a representative section of implanted dermis (Fig. 6) and the surface of the dermis was re-epithelialized partially. Moreover, healthy revascularization was found in the implanted dermis. Minimal inflammatory cells were found in the graft.

Similar results were found for MH-ADM in a study by Capito et al. (2012) where the integrative properties of MH-ADM (also described in the study as DermACELL) and three other ADMs (AlloDerm™, DermaMatrix™ (Synthes, Inc., West Chester, PA), and Integra™ (Integra LifeSciences Corporation, Plainsboro, NJ)) were compared in a rat model. Tissue revascularization, recellularization, and integration were evaluated at four time points ranging from 7 to 42 days. Out of the four ADMs evaluated, MH-ADM had the highest cell density measured at 300, 600, and 900 µm from the blood-vessel graft interface at all time points except Day 42. This difference was statistically significant for many of the time points and distances. Furthermore, MH-ADM had the highest amount of cellular infiltration at all time points, which was significantly greater than two of the other ADMs. Additionally, MH-ADM had a statistically significant greater amount of blood vessel formation in the tissue than the other three ADMs at Day 7 and still had a statistically significant greater amount than two other ADMs at Day 42. In all three objective evaluations, MH-ADM compared very favorably with the other three ADMs tested.

## Clinical applications of MH-ADM

Clinical applications of ADMs have been noted in orthopaedic surgeries, dental and craniomaxillofacial repairs, soft tissue reconstruction, and wound healing. Orthopaedic surgeons commonly use ADMs in soft tissue repair procedures to provide additional biomechanical strength and improve healing for rotator cuff repairs, especially for large and massive tears (Wong et al. 2010; Snyder and Bone 2007; Barber et al. 2008; Burkhead et al. 2007; Bond et al. 2008; Dopirak et al. 2007). In addition, ADM was applied to augment Achilles tendon for increased biomechanical strength, possible enhanced healing, and reduced return to activity times (Lee 2007, 2008).

**Fig. 7:**
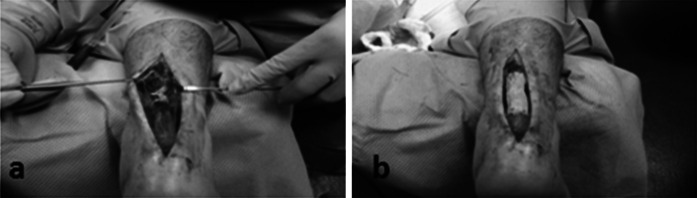
Use of MH-ADM to augment an Achilles tendon repair. Photo courtesy of Jeffrey Barton, DPM (Delaware Surgery Center, Dover, DE) showed before (**a**) and after (**b**) the augmentation

**Fig. 8:**
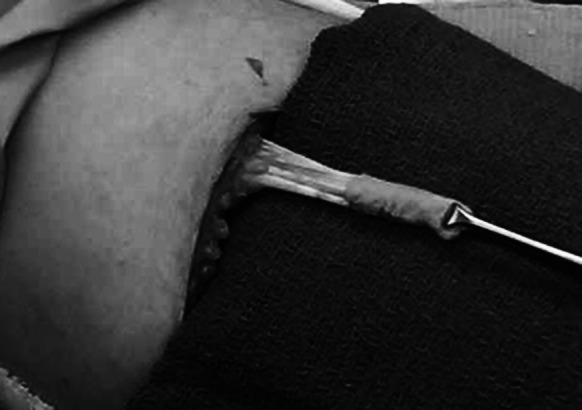
Use of MH-ADM to augment a biceps tendon repair. Photo courtesy of Raffy Mirzayan, MD, Los Angeles, CA

For rotator cuff repair, post-operative reports showed reduced pain and increased motion (Levenda and Sanders 2012). The authors described an arthroscopic technique for performing rotator cuff repair augmented with MH-ADM in a 10 patient case series study. At a post operative follow-up of 6 months to 1 year, 9 of 10 patients reported decreased pain and showed increased motion. The 10th patient was also progressing well until a fall, 3 months post-operative, tore the rotator cuff and a total shoulder arthroplasty was performed. The graft augmentation was found to be intact during the arthroplasty. In addition, MH-ADM was also used in repair and augmentation of Achilles tendon (Fig. 7), quadriceps, and biceps tendon (Fig. 8).

**Fig. 9:**
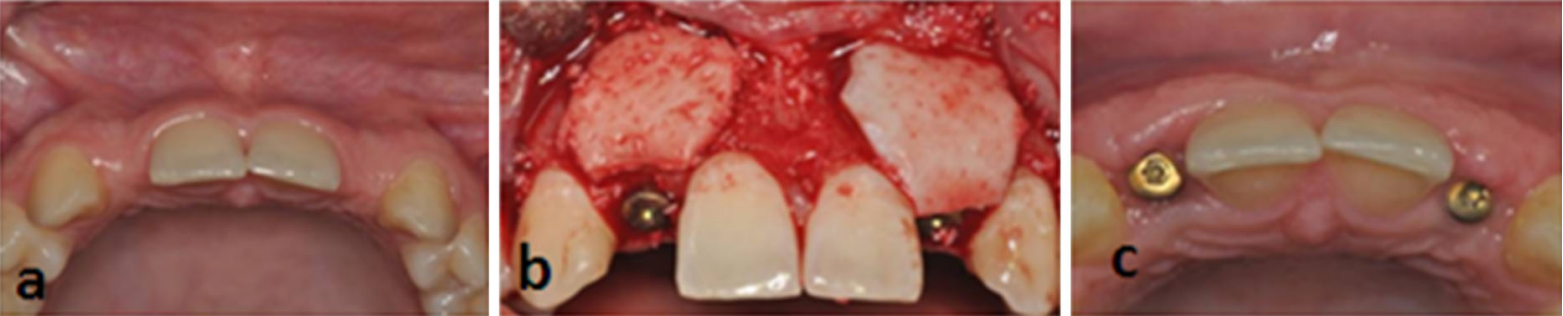
Use of MH-ADM in conjunction with cortical bone particulates to correct for the thin bone implant support and to increase the soft tissue profile in these areas. **a** Pre-surgery showing a thin bone ridge. **b** The application of MH-ADM in conjunction with implant bone and pins. **c** 4 weeks post-implant demonstrating increase in tissue profile and a smoothly healed gum line. The case photos are courtesy of Paul Rosen, DMD, MS (Yardley, PA)

**Fig. 10:**
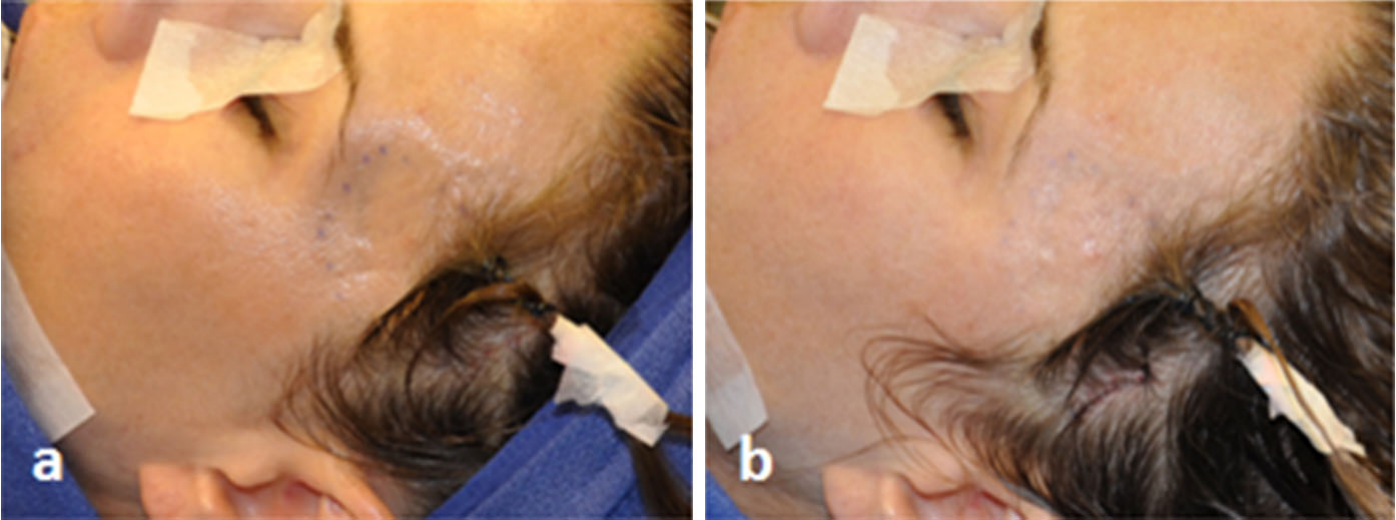
Use of MH-ADM to repair a temporal depression. **a** Pre-surgery showing the temporal depression. **b** The depression smoothing following an incision in the hair line and insertion of MH-ADM. The case photos are courtesy of Barry Eppley, MD (Indianapolis, IN)

Bone resorption following tooth extraction requires immediate ridge augmentation to prevent further resorption affecting the placement of dental implants (Wallace 2013). MH-ADM was able to facilitate guided bone regeneration (GBR) (Wallace 2013) and soft tissue alveolar ridge augmentation (Al-Hamdan 2011) without the second harvest site morbidity associated with autografts. In another example of dental application (Fig. 9), MH-ADM has been utilized in conjunction with cortical bone particulate to correct for thin bone implant support. After 4 weeks, there was an increase in tissue profile and the gum line had healed smoothly. Additionally, MH-ADM was used to repair temporal depressions by smoothing over depressions and providing a natural appearance (Fig. 10).

**Fig. 11:**
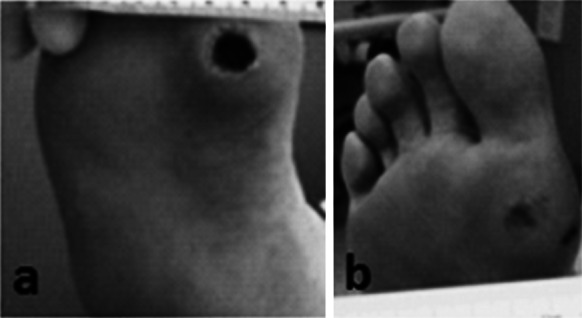
Use of MH-ADM for wound repair of a diabetic foot ulcer. **a** 47 y.o. female presented with a Wagner Grade 2 non-healing diabetic ulcer on the plantar first medial head. **b** The patient went on to a successful outcome with a single application of MH-ADM as noted by the substantial healing at week 12 (Yonehiro et al. 2013: reproduced with permission of the authors)

ADMs have been commonly used for treatment of acute and chronic wounds. Non-healing, diabetic ulcers of the lower extremities, particularly the foot, can be treated with an ADM to achieve complete healing and integration while avoiding second site morbidity associated with autografts (Winters et al. 2008; Randall et al. 2008). Yonehiro et al. (2013) successfully treated diabetic foot ulcers using MH-ADM with a substantial wound healing rate of 83 % in their evaluation of 11 patients with 12 non-healing diabetic ulcers. In one of the cases, a 47 year old female presenting with a Wagner Grade 2 non-healing, diabetic ulcer on the plantar first medial head (Fig. 11a) was treated with MH-ADM. The patient went on to a successful outcome with substantial healing at week 12 after a single application of MH-ADM (Fig. 11b). A burn wound treated using MH-ADM demonstrated healing, reduced scarring, and apparent revascularization and recellularization (Chen et al. 2012). In this case study, a patient who suffered from 2nd and 3rd degree burns was initially treated with antibiotics and dressings and over the next 3 years experienced significant scarring and a corresponding restricted range of motion. Following this period, the scar tissue was excised and MH-ADM was applied, and then overlaid with split thickness skin grafts. At 30 days post-operation, the wound bed lacked significant scarring and appeared to have rapid revascularization and recellularization. At 6 months post-operation, the wound healed with significantly less scarring and the patient’s range of motion dramatically improved.

**Fig. 12:**
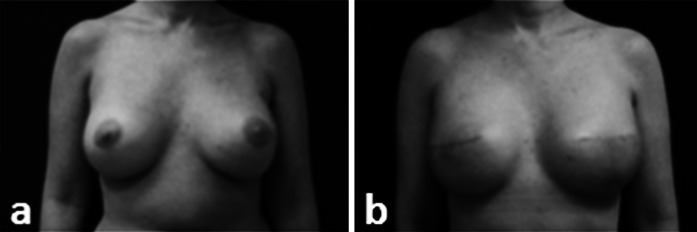
Use of MH-ADM for breast reconstruction. **a** pre-operative and **b** post-operative. In this case, a 46 y.o patient received a bilateral mastectomy and advanced to the 2nd stage repair. Her expanders were filled to the full 550 cc and replaced with a 700 cc silicone implant 16 weeks following mastectomy (Vashi 2014: reproduced with permission of the author)

**Fig. 13:**
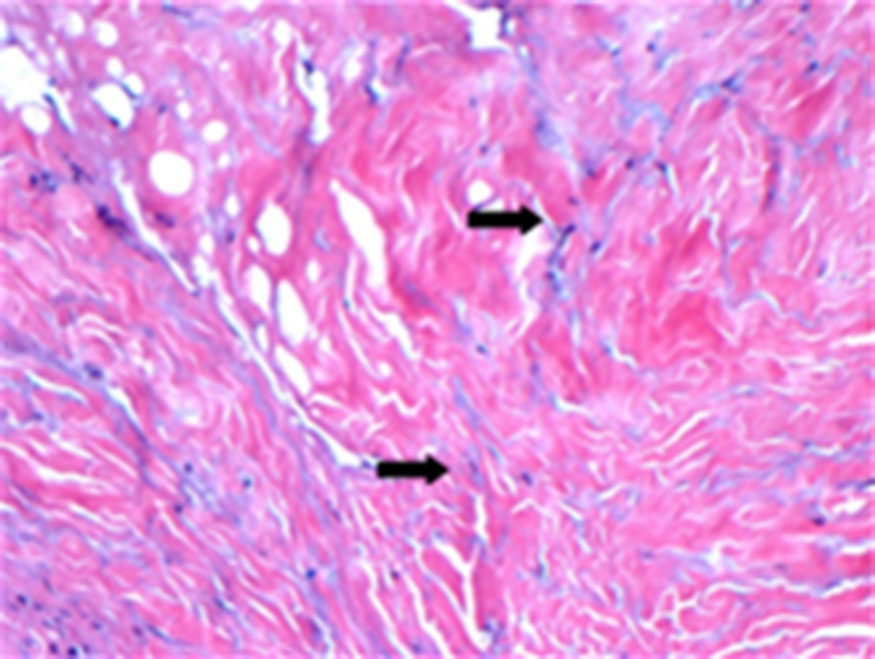
Hematoxylin and eosin staining of biopsy specimen following 16 weeks in situ placement of MH-ADM in the breast reconstruction surgery shown in Fig. 12. Note the intact ultrastructure and also evidence of cellular in-growth with apparent fibroblasts (arrows) at 10× magnification (data on file at LifeNet Health). (Color figure online)

Soft tissue reconstruction procedures of the breast and plantar heel also commonly utilize ADMs. Two-stage, primary, and revision breast reconstructions used ADM to extend the pectoralis muscle and improve cosmetic appearance while maximizing available skin and reducing reconstruction time (Sbitany et al. 2009; Salzberg 2006; Ortiz 2014). Using MH-ADM, a 46 year old woman underwent a bilateral mastectomy with immediate 2nd stage breast reconstruction (Fig. 12). Her expanders were filled to 550 cc and after 16 weeks following the mastectomy they were replaced with 700 cc permanent silicone implants. The histological analysis (Fig. 13) on biopsy tissue demonstrated that the MH-ADM was incorporated into the surrounding tissue. The Hematoxylin and Eosin staining of the biopsy specimen show clearly identifiable intact ultrastructure of extracellular matrix and fibroblast infiltration.

MH-ADM used in tissue replacement of the plantar heel achieved reduced pain involved with ambulation, particularly in weight-bearing areas of the heel (Mulder 2012). In this case series, MH-ADM successfully replaced missing tissue of the plantar heel in 3 patients who had previously lost nearly all of their plantar heel fat pads due to severe motor vehicle accidents. The first patient exhibited encouraging results with pain free ambulation at 6 weeks post-operation and there was continued patient satisfaction at a 3 months post-operative follow-up visit. At over 1 year post-operation, this patient remained pain free. The other two patients did not have long term follow-ups but their initial results were similar to the first patient, supporting the use of MH-ADM for treating plantar defects.

## Conclusions

As reviewed here, the MatrACELL process effectively removes cellular material, including DNA and immunogenic components, yielding an acelluar dermis, MH-ADM, which retains biomechanical strength and is biocompatible. Both preclinical and clinical results support the use of this allograft tissue in a myriad of clinical applications, including tendon augmentation, facial reconstruction, wound healing, soft tissue reconstruction, and dental procedures.
